
JOURNAL INFORMATION
==============================
NLM Title Abbreviation: ScientificWorldJournal
Journal ID: ScientificWorldJournal
NLM Journal ID: 101131163
Journal ID: TSWJ

The Scientific World Journal

ISSN: 2356-6140
EISSN: 1537-744X
Publisher: Wiley

ARTICLE INFORMATION
==============================
PMCID: PMC5974593
DOI: 10.1100/tsw.2003.28
Article ID: 425680
Article version: 1
Subjects: Book Review

Book Review "Early Prediction and Prevention of Child Abuse"

Merrick Joav *
National Institute of Child Health and Human Development, Division of Community Health, Zusman Child Development Center, Ben Gurion University, Beer-Sheva and Office of the Medical Director, Division for Mental Retardation, Ministry of Social Affairs, Jerusalem, Israel
*Joav Merrick: jmerrick@internet-zahav.netAcademic Editor: Joav Merrick

Publication date: 2003
Electronic publication date: 2003 Apr 28
Volume: 3
First page: 279
Last page: 280
Copyright: Copyright © 2003 Joav Merrick.
Copyright year: 2003
License: This is an open access article distributed under the Creative Commons Attribution License, which permits unrestricted use, distribution, and reproduction in any medium, provided the original work is properly cited.
License URL: https://creativecommons.org/licenses/by/3.0/

Keywords: child abuse, child neglect, prevention, early prediction

==============================
Book Review
*TheScientificWorldJOURNAL* (2003) 3, 279-280
ISSN 1537-744X; DOI 10.1100/tsw.2003.28

### Book Review — Early Prediction and Prevention of Child Abuse

Joav Merrick, M.D.
*National Institute of Child Health and Human Development, Division of Community Health, Zusman Child Development Center, Ben Gurion University, Beer-Sheva and Office of the Medical Director, Division for Mental Retardation, Ministry of Social Affairs, Jerusalem, Israel*

**Email:** jmerrick@internet-zahav.net

*Received April 11, 2003; Accepted April 11, 2003; Published April 28, 2003*

***Early Prediction and Prevention of Child Abuse. A Handbook.***

**Edited by Kevin D. Browne (University of Birmingham, U.K.), Helga Hanks (St. Jamem's University Hospital, Leeds, U.K.), Peter Stratton (University of Leeds, U.K.), and Catherine Hamilton (University of Birmingham, U.K.).**

**John Wiley & Sons, Chichester. 2002, 382 p. Price: 55.00 US$ (softcover).**

**ISBN 0-471-49122-5**

**KEYWORDS:** child abuse, child neglect, prevention, early prediction

**DOMAINS:** child health and human development, behavior, behavioral psychology, human rights, psychiatry, social psychology, sociology, clinical medicine, medical care, nursing

This book is a new and updated version of a book published in 1988 on the prevention of child abuse and neglect in the early pioneering days of child abuse research. The editors are an all British team from the Universities of Birmingham and Leeds with 38 contributors from the U.K., U.S., and Australia.

There are 21 chapters divided into 4 sections: prevalence and prediction; primary and secondary prevention; tertiary prevention; and working with offenders.

In Section I it is interesting to note that over the last 25 years, when several countries started to collect data on child abuse cases on a regular basis, some evidence has been observed that the prevalence of child abuse has decreased. This positive information may be attributed to the focus on preventive public health approaches that many countries have undertaken after too many famous cases of fatal child abuse covered by the media. Section II is influenced by the 1979 book by Uri Bronfenbrenner on the ecology of human development; it is still very relevant today. In

©2003 with author.

Sections III and IV, the issue is tertiary prevention with a focus not only on the child and family, but also the offenders.

Much has happened since 1988, in many countries, for better prevention, intervention, and a switch to comprehensive child health policies, but there is still a lot of work to be done.

I especially liked the chapter by David Olds et al. from Colorado on his last 24 years of research on nurse home visiting in the prenatal and infant period. Here you will find the evidence base for early intervention of the model of the nurse visitor coming to the home during pregnancy and continuing during infancy. This model was implemented in Denmark in 1936 on a national level and used by nearly 100% of all pregnant women in Denmark. David Olds et al., in his studies in the Appalachian region of New York State (the Elmira study) and Memphis, Tennessee, has shown that early intervention has functional and economic benefits with the greatest impact on families at high risk. Their Nurse Family Partnership now functions in 24 states and over 200 local communities in the U.S.

This book has value for a range of professionals (both in research and clinical practice), policy makers, and managers working in the field of child protection, child and human development.

**This article should be referenced as follows**:

Merrick, J. (2003) Book review — Early prediction and prevention of child abuse. *TheScientificWorldJOURNAL* **3**, 279–280.
